# The Risks of Anesthesia

**Published:** 1896-03

**Authors:** 


### The Risks of Anæsthesia.

It is stated that sixty-one deaths have occurred within the past year in the United Kingdom, of which fifty-two were from the administration of chloroform. This would be a fearful indictment against the use of that anæsthetic if we only knew what was the relative proportion of the patients submitted to its influence and to the influence of other anæsthetics. In other words, if the number of chloroform cases were fifty-two times the number of nitrous oxide cases chloroform would be no more dangerous, although it might have caused fifty-two deaths for one death caused by the latter anæsthetic.—*Med. Press and Circular.*

